# Essential Oil Composition and Biological Activities of *Hyptis eriocephala*, an Aromatic Lamiaceae Species from Southern Ecuador

**DOI:** 10.3390/plants15152407

**Published:** 2026-08-06

**Authors:** Diana Guaya, Ana Cristina Hernández, Vladimir Morocho

**Affiliations:** 1 Departamento de Química y Producción, Universidad Técnica Particular de Loja (UTPL), Calle París s/n y Praga, Loja 110107, Ecuador; 2 Carrera de Bioquímica y Farmacia, Universidad Técnica Particular de Loja (UTPL), Calle París s/n y Praga, Loja 110107, Ecuador; cris_hernadez12@hotmail.com; 3 Independent Researcher, Loja 110102, Ecuador; vladimirmorocho@gmail.com

**Keywords:** *Hyptis eriocephala*, essential oil, hydrocarbon sesquiterpenes, α-copaene, ABTS radical scavenging, cholinesterase inhibition

## Abstract

*Hyptis eriocephala* is an aromatic Lamiaceae species from southern Ecuador whose essential oil has not previously been characterized. To establish an initial phytochemical and biological baseline, aerial parts collected at Villonaco were subjected to three independent steam-distillation runs, yielding 0.13 ± 0.04% (*w*/*w*) essential oil. Volatile constituents were tentatively assigned by GC–MS using concordant EI mass-spectral and linear retention-index evidence, whereas relative composition was determined by GC–FID peak-area normalization without an internal standard, calibration curves, or response correction factors. Sixty-five constituents accounted for 96.81% of the total GC–FID peak area. The relative profile was dominated by hydrocarbon sesquiterpenes (67.88%), followed by hydrocarbon monoterpenes (24.36%), with α-copaene (28.20 ± 0.24%), germacrene D (9.29 ± 0.09%), α-pinene (6.98 ± 0.03%), α-cubebene (6.70 ± 0.06%), δ-cadinene (6.03 ± 0.07%), limonene (5.44 ± 0.05%), (E)-caryophyllene (5.31 ± 0.05%), and β-phellandrene (4.96 ± 0.02%) were the principal constituents. The oil showed a narrow antibacterial response, with MIC values of 250 µg/mL against *Enterococcus faecium* ATCC 27270 and 2000 µg/mL against *Enterococcus faecalis* ATCC 19433. Radical-scavenging activity was assay-dependent: activity was measurable in the ABTS assay (SC_50_ = 76.87 ± 1.05 µg/mL; TEAC = 30.06 ± 1.16 µM TE/g EO), whereas 50% DPPH scavenging was not reached at 8000 µg/mL. The oil preferentially inhibited BuChE (IC_50_ = 125.3 ± 1.03 µg/mL) over AChE (IC_50_ = 385.9 ± 1.02 µg/mL). These findings establish a preliminary phytochemical and biological baseline for the Villonaco material but do not demonstrate a species-wide chemotype, mechanism of action, or therapeutic potential.

## 1. Introduction

Medicinal and aromatic plants are important sources of bioactive natural products and have contributed to the development of pharmaceutical, cosmetic, and agroindustrial applications [1,2,3,4,5]. Among their specialized metabolites, essential oils are of particular interest because their volatile constituents may exhibit antimicrobial, antioxidant, anti-inflammatory, and enzyme-modulating activities [6,7]. Their chemical profiles may also provide comparative phytochemical information among species and populations; however, such profiles should not be interpreted as evidence of chemotypes, taxonomic differentiation, or ecological adaptation unless their stability is demonstrated across multiple populations, seasons, and environmental conditions.

Ecuador has a longstanding tradition of medicinal plant use [8]. Nevertheless, recent investigations continue to document previously uncharacterized volatile metabolites and biological properties in Ecuadorian aromatic species [9]. Within this flora, Lamiaceae is especially relevant because its species possess glandular trichomes involved in the biosynthesis, secretion, and storage of volatile metabolites, particularly mono- and sesquiterpenes [10,11]. Consequently, the family has received sustained attention in essential oil chemistry, pharmacognosy, biological screening, and comparative phytochemical studies [12,13].

The genus *Hyptis* comprises approximately 300 species distributed mainly throughout tropical and subtropical regions of the Americas [14]. Essential oils reported for this genus exhibit substantial interspecific and intraspecific variability, with profiles dominated by monoterpenes, hydrocarbon sesquiterpenes, or oxygenated terpenoids [15,16,17]. Their composition may vary according to species, geographic origin, plant organ, phenological stage, and environmental conditions [18,19]. Therefore, the phytochemical characterization of an unstudied *Hyptis* species should not be limited to determining whether its oil is monoterpene- or sesquiterpene-rich, because both patterns have already been described within the genus. Instead, its distinctiveness should be evaluated through the identity and relative distribution of its major constituents in comparison with previously characterized species.

*Hyptis eriocephala* is an aromatic species native to South America and distributed in Andean and inter-Andean regions of Ecuador. Ethnobotanical records identify the species by the Kichwa name Aya tukana and document its traditional use by communities from the Ecuadorian Sierra, particularly in Loja. Its leaves and young-shoot sap are used to treat “mal aire,” decoctions are employed in body washing to reduce fever, and flower sap is used for nervous conditions [8]. Although these traditional applications do not establish that the essential oil is responsible for the reported effects, they demonstrate the ethnobotanical relevance of the species and support its prioritization as a chemically unexplored aromatic plant from southern Ecuador.

To the best of our knowledge, the essential oil of *H. eriocephala* has not previously been characterized. In contrast, the volatile composition of several other *Hyptis* species, including *H. spicigera*, *H. suaveolens*, *H. emoryi*, *H. conferta*, *H. crenata*, *H. fruticosa*, *H. goyazensis*, and *H. floribunda*, has been investigated [20]. The absence of information on *H. eriocephala* prevents assessment of whether its volatile composition conforms to previously described patterns within the genus or represents a distinctive chemical profile. It is also unknown whether its essential oil exhibits broad biological activity or differential responses among microbial strains, radical-scavenging systems, and cholinesterase enzymes.

Accordingly, this study addressed whether the essential oil of *H. eriocephala* collected in southern Ecuador exhibits a volatile composition distinguishable from those reported for other *Hyptis* species, and whether it produces measurable, assay-dependent in vitro biological responses. We hypothesized that the oil would exhibit a distinctive terpenoid distribution and differential antimicrobial, radical-scavenging, and cholinesterase-inhibitory activities. To test this hypothesis, the volatile constituents were tentatively assigned by GC–MS, their relative peak-area composition was determined by GC–FID, and the biological activities of the essential oil were evaluated using complementary in vitro assays. Because the study examined material collected from a single location during one collection period, the findings are interpreted as an initial phytochemical and biological baseline. This baseline may support future comparative studies addressing geographic, seasonal, and phenological variation, as well as activity-guided investigations of potentially relevant constituents. However, the present results do not establish a species-wide chemotype or provide evidence of ecological adaptation.

## 2. Results and Discussion

### 2.1. Essential Oil Yield and Physicochemical Properties

The essential oil obtained from the aerial parts of *Hyptis eriocephala* was pale yellow and recovered in an average yield of 0.13 ± 0.04% (*w*/*w*), calculated on a dry weight basis (Table 1). Although low, this yield falls within the variability reported for the genus *Hyptis*. Higher yields have been reported for *H. crenata* (1.1–3.1%), *H. suaveolens* (0.25–0.65%, depending on the growth stage), and *H. dilatata* (1.04%), indicating that essential oil productivity within the genus depends on species, developmental stage, and environmental conditions [19,21,22]. The Villonaco material was collected at high altitude on an exposed Andean ridge. Environmental conditions associated with such settings can modulate secondary metabolism in aromatic plants and may have contributed to the yield and chemical profile observed here [18].

The observed yield should also be interpreted in relation to the use of wild-collected aerial parts, the flowering stage, the seven-day drying period, and the ecological conditions of the collection site [18,23]. Although the low yield may constrain practical essential oil recovery, it does not diminish the value of this first phytochemical characterization. Comparative studies involving different plant organs, phenological stages, seasons, and locations are needed to determine the stability of oil productivity and composition [24].

The refractive index of the essential oil was determined as n^20^_D_ = 1.4875 ± 0.0020. This value is higher than that reported for *Hyptis suaveolens* essential oil (1.4731), illustrating physicochemical variability within the genus [25]. Because refractive index reflects the bulk properties of the mixture, it cannot be attributed to individual constituents.

The specific optical rotation of the essential oil was measured as [α]^20^_D_ = –11.05 ± 0.03, indicating an overall levorotatory character under the measurement conditions. This bulk parameter does not establish the enantiomeric distribution of individual mono- or sesquiterpenes [26]. Enantioselective GC analysis would therefore be required to characterize the stereochemical composition of the oil.

### 2.2. Chemical Composition of the Essential Oil

The volatile profile of Hyptis eriocephala essential oil was analyzed by GC–MS, whereas its relative composition was determined by GC–FID peak-area normalization using a non-polar TR-5MS capillary column. Compound assignments were based on concordant EI mass-spectral and LRI evidence. Sixty-five volatile constituents were tentatively assigned, accounting for 96.81% of the total GC–FID peak area. The three essential oils obtained from the independent distillations were analyzed separately, and the reported relative composition values are expressed as mean ± SD across three independent extraction replicates (n = 3).

A non-polar TR-5MS capillary column is widely used for essential oil profiling; however, structurally related terpenes may co-elute in complex sesquiterpene-rich matrices. In the present study, agreement between experimental and reference LRIs, together with concordant EI mass-spectral matches, supported high-confidence tentative assignments for the major constituents [27,28,29].

The relative GC–FID peak-area profile was dominated by hydrocarbon sesquiterpenes (67.88%), followed by hydrocarbon monoterpenes (24.36%), oxygenated sesquiterpenes (2.49%), other compounds (1.73%), and oxygenated monoterpenes (0.35%). These values represent relative peak-area percentages rather than absolute concentrations and define a sesquiterpene-rich chemical profile for the Villonaco material (Table 2).

**Figure 1:**
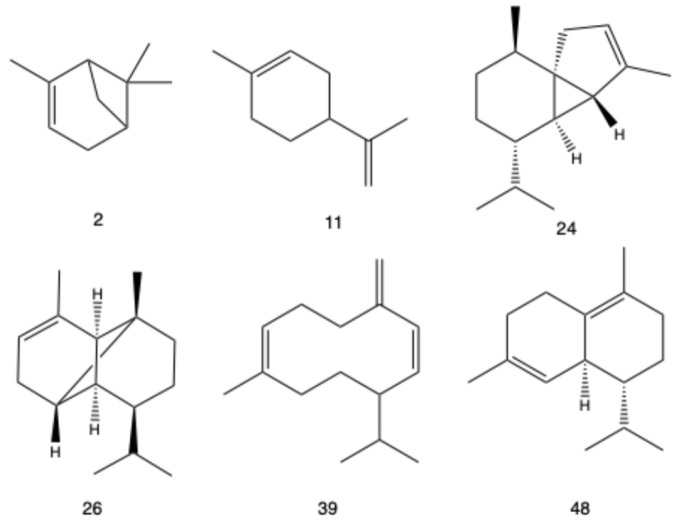
Chemical structures of selected major constituents tentatively assigned in *Hyptis eriocephala* essential oil by GC–MS: (2) α-pinene, (11) limonene, (24) α-cubebene, (26) α-copaene, (39) germacrene D, and (48) δ-cadinene. Compound numbers correspond to Table 2.

The major constituents of the oil were α-copaene (28.20 ± 0.24%), germacrene D (9.29 ± 0.09%), α-pinene (6.98 ± 0.03%), α-cubebene (6.70 ± 0.06%), δ-cadinene (6.03 ± 0.07%), limonene (5.44 ± 0.05%), and (E)-caryophyllene (5.31 ± 0.05%) (Figure 1). The predominance of α-copaene, together with the substantial contributions of germacrene D, δ-cadinene, and (E)-caryophyllene, distinguishes the Villonaco material from essential oil profiles reported for other members of the genus.

**Figure 2:**
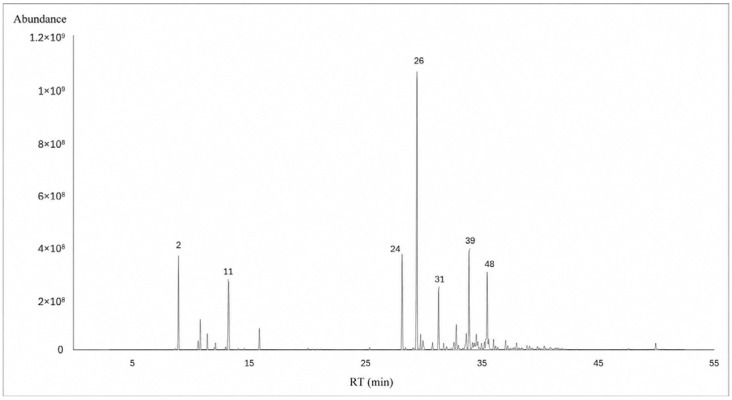
GC–MS chromatogram of *Hyptis eriocephala* essential oil acquired using a non-polar TR-5MS capillary column. Labeled peaks correspond to the major constituents tentatively assigned as (2) α-pinene, (11) limonene, (24) α-cubebene, (26) α-copaene, (31) (E)-caryophyllene, (39) germacrene D, and (48) δ-cadinene; peak numbers correspond to Table 2.

Figure 2 presents the GC–MS chromatogram of the essential oil of *H. eriocephala* obtained on a non-polar TR-5MS capillary column. The profile shows a complex volatile mixture with well-defined peaks corresponding to the principal constituents listed in Table 2. Peak assignments were supported by agreement between experimental LRIs and reference values and by concordant mass-spectral library matches [27,28].

Essential oils from Hyptis species are generally described as mixtures mainly composed of mono- and sesquiterpenes; however, marked differences in the relative abundance of key constituents support comparative phytochemical assessment among species [20]. In this context, the chemical profile of the Villonaco material differs from those reported for several previously investigated taxa of the genus. For instance, the essential oil of *H. suaveolens* has been reported to be dominated by β-caryophyllene (33.9%), germacrene D (25.4%), α-humulene (8.3%), and germacrene B (8.2%), indicating a sesquiterpene-rich profile, but with major constituents clearly different from those identified in the present study [30]. Likewise, *H. crenata* exhibits distinct chemotypes, one characterized by 1,8-cineole (31.0%), α-pinene (13.6%), (E)-caryophyllene (7.8%), and β-pinene (7.6%), and another dominated by 1,8-cineole (17.4–23.5%), α-pinene (15.7–23.5%), β-pinene (10.5–13.4%), and limonene (8.5–9.7%) [21]. Similarly, *H. dilatata* showed marked intraspecific variability, with one profile dominated by limonene (72.6%), myrcene (11.5%), and p-cymene (10.3%), and another characterized by camphor (25.5%), α-pinene (25.4%), 1,8-cineole (18.8%), β-pinene (12.0%), and limonene (5.9%) [22]. In addition, *H. pectinata* has been reported to contain approximately 90% sesquiterpenes, with β-caryophyllene (17.66%) and calamusenone (36.08%) as major constituents in different chemotypes, while caryophyllene oxide was consistently identified as a secondary compound [31].

Compared with essential oil profiles reported for other *Hyptis* species, the Villonaco material was distinguished by the predominance of α-copaene and by the relative distribution of its principal mono- and sesquiterpenes. This combination defines a distinctive sesquiterpene-rich chemical profile for the material analyzed and provides a baseline for future comparisons across locations, seasons, phenological stages, and years.

### 2.3. Biological Activity

The biological activity of *H. eriocephala* essential oil was evaluated using antimicrobial, radical-scavenging, and cholinesterase-inhibition assays.

#### 2.3.1. Antimicrobial Activity

The essential oil showed a narrow antibacterial response. The strongest inhibition was observed against *Enterococcus faecium* ATCC 27270, with a MIC of 250 µg/mL, whereas the activity against *Enterococcus faecalis* ATCC 19433 was substantially weaker, with a MIC of 2000 µg/mL (Table 3). No inhibition was detected against the remaining bacterial or fungal strains at the highest concentration tested. These findings indicate a strain-dependent response largely restricted to *Enterococcus* species rather than broad-spectrum antimicrobial activity.

Antimicrobial activity reported within the genus *Hyptis* varies considerably among species and experimental conditions. Essential oils of *H. suaveolens* have shown variable antibacterial and antifungal effects, including changes associated with the phenological stage of the plant [19,32]. Broader antimicrobial profiles have been reported for *H. atrorubens*, which inhibited several bacterial and fungal strains, and for *H. pectinata*, which showed greater activity against Gram-positive bacteria and yeasts [33,34]. In contrast, *H. capitata* essential oil did not inhibit *Staphylococcus aureus* or *Escherichia coli* under the conditions evaluated [35]. These differences indicate that antimicrobial activity within the genus cannot be inferred solely from taxonomic affiliation or from the general presence of mono- and sesquiterpenes.

Selective or weak antimicrobial responses have also been reported for other terpene-rich essential oils from Ecuador. *Hedyosmum purpurascens* essential oil exhibited limited activity against the microorganisms evaluated, whereas the leaf and fruit oils of *Zanthoxylum mantaro* produced strain-dependent responses [36,37]. These comparisons place the limited antimicrobial spectrum of *H. eriocephala* within the variability observed for chemically complex essential oils but do not explain the inhibition detected against *E. faecium*.

Because only the complete essential oil was evaluated, the observed activity cannot be attributed to α-copaene or to any other individual constituent. A relevant comparison is provided by *H. colombiana*, whose antibacterial activity varied between collection periods despite the recurrent presence of germacrene D and β-caryophyllene [38]. This observation illustrates that compositional similarity or the presence of major sesquiterpenes does not necessarily result in comparable antimicrobial activity. Potential additive, synergistic, or antagonistic interactions among constituents therefore remain untested hypotheses, since no fractionation, isolated-compound, or recombination experiments were performed in the present study [39].

Accordingly, the inhibition of *E. faecium* should be interpreted as preliminary evidence of a narrow, strain-dependent in vitro antibacterial response rather than as a general antimicrobial property of *H. eriocephala*. Evaluation of additional *Enterococcus* isolates, time–kill assays, activity-guided fractionation, isolated-compound testing, and recombination experiments would be required to establish the reproducibility and chemical basis of this activity.

#### 2.3.2. Antioxidant Activity

Under the experimental conditions used, *H. eriocephala* essential oil exhibited radical-scavenging activity in the ABTS assay, with an SC_50_ value of 76.87 ± 1.05 µg/mL and a TEAC value of 30.06 ± 1.16 µM TE/g EO. In contrast, 50% DPPH scavenging was not reached at the highest concentration tested (8000 µg/mL); therefore, an SC_50_ value was not extrapolated (Table 4). These results indicate an assay-dependent response rather than broad antioxidant activity.

The contrasting ABTS and DPPH results should be interpreted cautiously because the two assays differ in radical chemistry, reaction medium, kinetics, and accessibility of the tested constituents. Consequently, they do not necessarily provide equivalent responses for complex volatile mixtures [40]. However, the present data do not establish which of these factors predominated, nor do they support attribution of the observed response to hydrocarbon sesquiterpenes, minor oxygenated constituents, or a specific electron- or hydrogen-transfer mechanism.

Variable antioxidant responses have also been reported within the genus *Hyptis*. *H. suaveolens* essential oil showed activity in both DPPH and ABTS assays, while subsequent analyses demonstrated that its response varied according to the plant growth stage [19,32]. In contrast, *H. capitata* exhibited limited DPPH scavenging activity [35]. Studies involving ethanolic extracts of *H. conferta*, *H. dilatata*, *H. mutabilis*, and *H. suaveolens* reported associations between antioxidant activity and total phenolic content [41]. Similarly, investigations of *H. colombiana* showed marked chemical differences between sesquiterpene-rich essential oils and phenolic-rich hydroalcoholic extracts [42]. These studies demonstrate that antioxidant responses within the genus depend on species, phenological stage, evaluated fraction, and assay conditions. Nevertheless, results obtained from polar extracts should not be directly extrapolated to essential oils, and these comparisons do not identify the constituents responsible for ABTS response of *H. eriocephala*.

A comparable pattern of measurable ABTS activity without a calculable DPPH SC_50_ has been reported for other Ecuadorian terpene-rich essential oils. The leaf and fruit oils of *Zanthoxylum mantaro* showed ABTS activity but did not reach 50% DPPH scavenging at the highest concentration tested, while *Hedyosmum purpurascens* essential oil was inactive in DPPH but showed ABTS radical-scavenging activity [36,37]. These comparisons indicate that the response observed for *H. eriocephala* is not unique among terpene-rich essential oils, although they do not establish a common chemical or mechanistic explanation.

Accordingly, the present findings should be interpreted as preliminary evidence of radical-scavenging activity under the specific conditions of a cell-free ABTS assay. The essential oil should not be described as broadly antioxidant, and the absence of a measurable DPPH SC_50_ should not be assigned to a particular compositional or reaction mechanism without direct experimental evidence. Complementary lipid-phase and cellular oxidative-stress assays would be required to determine the reproducibility and biological relevance of the observed response.

#### 2.3.3. Cholinesterase Inhibitory Activity

**Figure 3:**
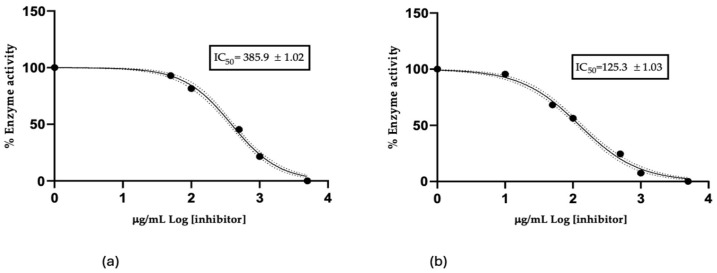
Concentration–response curves for inhibition of (**a**) acetylcholinesterase (AChE) and (**b**) butyrylcholinesterase (BuChE) by *Hyptis eriocephala* essential oil using the modified Ellman assay. Residual enzyme activity (%) is plotted against the logarithm of the final essential oil concentration (µg/mL). One experimental run was performed per enzyme; the three independently distilled oils were evaluated separately, each in technical triplicate. IC_50_ values are expressed as mean ± SD across the three independent extraction replicates (n = 3). Dots represent the experimental data, solid lines represent the fitted four-parameter logistic curves, and dashed lines indicate the 50% residual enzyme activity level used to estimate the IC_50_ values.

The essential oil of *H. eriocephala* inhibited both acetylcholinesterase (AChE) and butyrylcholinesterase (BuChE), with IC_50_ values of 385.9 ± 1.02 µg/mL and 125.3 ± 1.03 µg/mL, respectively (Figure 3). These values are expressed as mean ± SD across the three independently distilled essential oil samples evaluated separately (n = 3 extraction replicates). The approximately threefold lower IC_50_ value for BuChE indicates a preferential inhibitory response toward this enzyme under the experimental conditions used. This difference should be interpreted as in vitro enzyme selectivity rather than as evidence of therapeutic potential.

Comparative data from other terpene-rich essential oils provide context for the magnitude and selectivity of this response. *Pinus heldreichii* subsp. *leucodermis* essential oil inhibited AChE and BuChE with IC_50_ values of 51.1 and 80.6 µg/mL, respectively [43]. *Araucaria brasiliensis* essential oil showed IC_50_ values of 225.3 ± 24.2 µg/mL for AChE and 95.7 ± 20.8 µg/mL for BuChE, displaying a preferential BuChE inhibitory profile broadly similar to that observed for *H. eriocephala* [44]. In contrast, the flower and leaf essential oils of *Lepechinia paniculata* inhibited both enzymes at concentrations below 50 µg/mL and were therefore more potent than the oil evaluated in the present study [45]. Preferential BuChE inhibition has also been reported for several Lamiaceae essential oils, including peppermint, rosemary, sage, and different oregano chemotypes [46]. Accordingly, *H. eriocephala* showed lower inhibitory potency than the *Pinus* and *Lepechinia* oils but an enzyme-preference pattern comparable to that of *Araucaria* and some Lamiaceae oils. Nevertheless, differences in enzyme sources, assay conditions, concentration ranges, and curve-fitting procedures limit direct quantitative ranking among studies.

Some volatile constituents detected in *H. eriocephala* essential oil, including β-phellandrene and (E)-caryophyllene, have shown cholinesterase inhibitory activity in previous isolated-compound or essential oil studies [43,47]. However, only the complete essential oil was evaluated here; consequently, the observed inhibition cannot be attributed to α-copaene or to any other individual constituent. Studies on *Salvia* and other essential oils have shown that the activity of whole oil may differ from that of its isolated major constituents [47,48]. Such findings demonstrate the limitations of predicting biological activity solely from chemical composition but do not prove synergistic interactions in *H. eriocephala*.

Potential additive, synergistic, or antagonistic effects among the volatile constituents therefore remain untested hypotheses, since no fractionation, isolated-compound, or recombination experiments were performed [39,47,48]. Overall, the results provide preliminary evidence of preferential in vitro BuChE inhibition. Activity-guided fractionation, evaluation of isolated constituents, recombination assays, mechanistic studies, and cytotoxicity testing would be required before the chemical basis or pharmacological relevance of this response can be established.

## 3. Materials and Methods

### 3.1. General Information

The chemical analysis of the essential oil was carried out using a Trace 1310 gas chromatograph coupled to an ISQ 7000 single-quadrupole mass spectrometer and equipped with a flame ionization detector (FID) (Thermo Fisher Scientific, Waltham, MA, USA). Compound identification by GC–MS and relative composition analysis by GC–FID were performed using a non-polar TR-5MS capillary column (5% phenyl-methylpolysiloxane; 30 m × 0.25 mm internal diameter × 0.25 µm film thickness), purchased from Thermo Fisher Scientific (Waltham, MA, USA). Helium was used as the carrier gas for all chromatographic analyses (Indura, Guayaquil, Ecuador). HPLC-grade dichloromethane (Sigma-Aldrich, St. Louis, MO, USA) was used for sample dilution, and a homologous series of n-alkanes (C9–C22) (ChemService, West Chester, PA, USA) was used for the calculation of linear retention indices. The physical properties of the essential oil were determined using an AbbE refractometer (Boeco, Germany) for refractive index measurements and a Hanon P81 automatic polarimeter (Advanced Technology Group Co., Ltd., Jinan, Shandong, China) for optical rotation.

For the antimicrobial assay, dimethyl sulfoxide (DMSO) was used as solvent and negative control. Mueller–Hinton broth and Sabouraud dextrose broth were purchased from DIPCO (Quito, Ecuador) and used for the bacterial and fungal assays, respectively. Ampicillin, ciprofloxacin, and amphotericin B were used as positive controls for Gram-positive bacteria, Gram-negative bacteria, and fungi, respectively. For the antioxidant assays, 2,2′-azino-bis(3-ethylbenzothiazoline-6-sulfonic acid) (ABTS), 2,2-diphenyl-1-picrylhydrazyl (DPPH), Trolox, and methanol (MeOH) were used. For the cholinesterase inhibitory assays, acetylcholinesterase (AChE), butyrylcholinesterase (BuChE), acetylthiocholine iodide, butyrylthiocholine iodide, 5,5′-dithiobis(2-nitrobenzoic acid) (DTNB), phosphate-buffered saline (PBS), Tris-HCl, magnesium chloride hexahydrate, DMSO, and donepezil hydrochloride were used. Unless otherwise stated, reagents and standards were purchased from Sigma-Aldrich (St. Louis, MO, USA).

In addition, absorbance measurements for the cholinesterase inhibitory assays were performed using a microplate spectrophotometer at 412 nm (EPOCH 2, BioTek, Winooski, VT, USA).

### 3.2. Plant Material Collection

**Figure 4:**
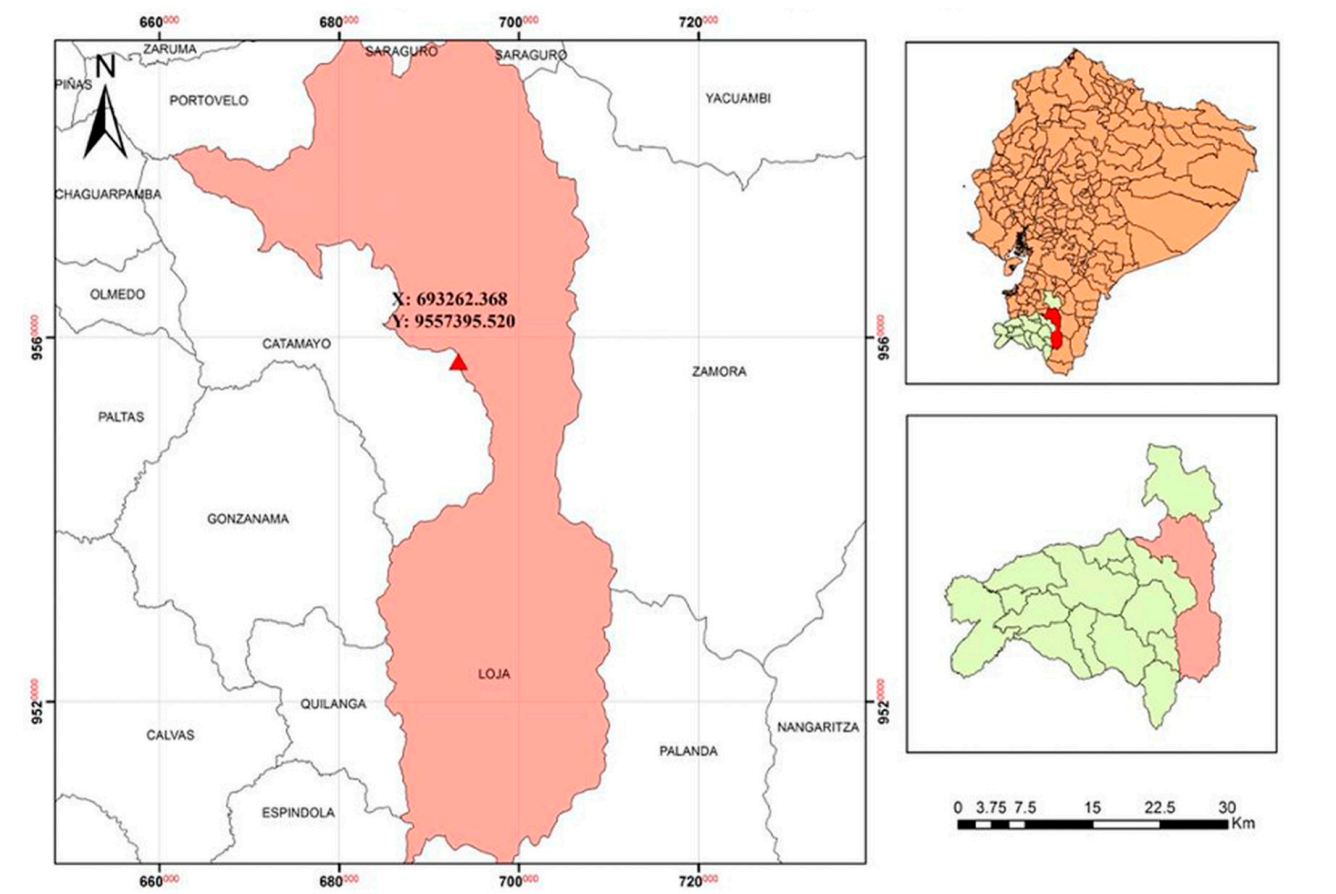
Location of the *Hyptis eriocephala* collection site at Villonaco Wind Farm, southern Ecuador (04°00′46.36″ S, 79°15′10.53″ W; approximately 2720 m a.s.l.). Aerial parts were collected at the flowering stage in September 2024.

The aerial parts of *Hyptis eriocephala* (Lamiaceae) were collected in September 2024 from the Villonaco Wind Farm area, located on the Andean ridge separating the cantons of Loja and Catamayo in southern Ecuador (04°00′46.36″ S, 79°15′10.53″ W; approximately 2720 m a.s.l.) (Figure 4). The collection site is located on an exposed Andean ridge characterized by marked seasonal rainfall and persistent winds. Long-term climatic data recorded for Villonaco during 1981–2020 indicate a mean temperature of 17.82 °C and mean monthly precipitation of 81.84 mm, equivalent to approximately 982 mm annually. Rainfall is concentrated mainly between November and April, whereas comparatively drier conditions occur from May to October [49].

The collected aerial parts consisted of leaves, young stems, and flowering inflorescences bearing floral buds and fully open flowers. All plant material was collected at the same flowering stage. Botanical identification was performed by Dr. Nixon Cumbicus, and a voucher specimen was deposited in the Herbarium of Universidad Técnica Particular de Loja (HUTPL 1430). After collection, the aerial parts were air-dried under shade for seven days in a well-ventilated area protected from direct sunlight. During the drying period, the ambient temperature was approximately 20–25 °C, with an estimated relative humidity of 50–60%. These conditions were used to reduce the moisture content while minimizing the loss or degradation of volatile constituents.

### 3.3. Essential Oil Extraction by Steam Distillation

Three independent steam-distillation runs were performed using separate batches of approximately 1100 g of dried aerial parts. The dried aerial parts were used whole and were not ground or milled before distillation. For each extraction replicate, water was placed in the lower chamber of the distillation unit, while the plant material was arranged on a perforated grid above the water without direct contact with the liquid phase. Steam generated in the lower chamber passed through the plant material, and the distillation was conducted for 4 h under continuous steam flow. The resulting vapors were condensed, and the essential oil was separated from the aqueous phase using a Florentine separator. The essential oil obtained from each distillation was dried over anhydrous sodium sulfate, transferred to a separate amber glass vial, hermetically sealed, and stored at 4 °C until analysis. Each distillation product was considered an independent extraction replicate. Essential Oil yield was calculated on a dry-weight basis and expressed as a percentage (w/w). The three essential oil samples were not pooled and were analyzed separately by GC–MS, GC–FID, and in the biological assays.

### 3.4. Determination of Physical Properties

The refractive index and specific optical rotation were determined separately for each of the three independently distilled essential oil samples. Each oil represented one independent extraction replicate, and the reported values are expressed as mean ± SD across the three extraction replicates (n = 3). The refractive index (n^20^_D_) of the essential oil was determined using an ABBE refractometer, according to ISO 280:1998 [50]. Measurements were performed using a single drop of essential oil, and values were reported as the mean of three independent determinations.

Specific optical rotation was measured using an automatic polarimeter (Hanon P81) in accordance with ISO 592:1998 guidelines [51]. For this purpose, 1 g of essential oil was diluted to 10 mL with dichloromethane. Measurements were carried out at 20 °C, and results were expressed as mean values obtained from three independent measurements.

### 3.5. Chemical Analysis of the Essential Oil

#### 3.5.1. Gas Chromatography–Mass Spectrometry (GC–MS)

The GC–MS analysis of the essential oil was performed using a Trace 1310 gas chromatograph coupled to an ISQ 7000 single-quadrupole mass spectrometer (Thermo Fisher Scientific, Waltham, MA, USA). Data acquisition and processing were performed using Chromeleon XPS software, version 7.2.10 (Thermo Fisher Scientific, Waltham, MA, USA). The analysis was performed on a non-polar TR-5MS capillary column (5% phenyl-methylpolysiloxane; 30 m × 0.25 mm internal diameter × 0.25 µm film thickness; Thermo Fisher Scientific, Waltham, MA, USA).

The mass spectrometer was operated in electron impact ionization mode at 70 eV, in full-scan mode. Helium of GC purity grade was used as the carrier gas at a constant flow rate of 1.0 mL/min. The injector temperature was maintained at 230 °C. A 1 µL aliquot of the essential oil sample, previously diluted 1:100 (*v*/*v*) in HPLC-grade dichloromethane, was injected in split mode, with a split flow of 100 mL/min. The essential oils obtained from the three independent distillations were analyzed separately by GC–MS. Thus, the three chromatographic profiles corresponded to three independent extraction replicates and not to repeated technical injections of a pooled essential oil sample. One GC–MS injection was performed for each independent extraction replicate.

The oven temperature program was as follows: the initial temperature was set at 50 °C and held for 3 min, then increased to 230 °C at a rate of 3 °C/min, and finally held at 230 °C for 3 min. The total chromatographic run time was approximately 63 min.

Compound assignments were based on two complementary criteria: (i) comparison of the experimental EI mass spectra with spectra contained in the NIST 17 Mass Spectral Library and with published spectral data; and (ii) comparison of the experimentally calculated linear retention indices with reference values reported for comparable low-polarity stationary phases [52,53]. LRIs were calculated according to the Van den Dool and Kratz method [54] using a homologous series of n-alkanes (C_9_–C_22_) analyzed under the same chromatographic conditions. Assignments were retained only when both the mass-spectral and LRI evidence were concordant. When structurally related compounds produced similar library matches, the experimental LRI was used to discriminate among candidate assignments [27]. For the major constituents, the absolute differences between calculated and reference LRIs ranged from 0 to 6 units. Together with concordant EI mass spectra, these results were considered to support high-confidence tentative assignments. Authentic standards and an additional stationary phase were not used; consequently, none of the compounds was considered unequivocally confirmed, and the possibility of co-elution or misassignment of structurally related sesquiterpenes cannot be completely excluded.

#### 3.5.2. Gas Chromatography–Flame Ionization Detection (GC–FID)

Relative peak-area composition was determined using the same gas chromatographic system equipped with a flame ionization detector. The chromatographic conditions were identical to those described for GC–MS. The relative percentage of each constituent was calculated by GC–FID peak-area normalization. No internal standard, calibration curves, or response correction factors were used; therefore, the reported values represent semi-quantitative relative peak-area percentages rather than absolute concentrations. Each essential oil obtained from the three independent distillations was analyzed separately by GC–FID. Mean relative percentages and standard deviations were calculated across the three independent extraction replicates (n = 3) and do not represent technical injections of a single pooled essential oil sample.

### 3.6. Biological Activity

The essential oils obtained from the three independent distillations were not pooled and were evaluated separately in all biological assays. Each distillation product was considered an independent extraction replicate (n = 3). Replicate wells or repeated measurements performed within each assay were treated as technical replicates and were averaged for each extraction replicate before data analysis. Accordingly, the reported biological results are based on the three independently distilled essential oil samples.

#### 3.6.1. Antimicrobial Assay

The antimicrobial activity of the essential oil of *H. eriocephala* was evaluated using the broth microdilution method, following the procedure described by Cartuche et al. [55], with minor modifications. The minimum inhibitory concentration (MIC) was determined against reference strains from the American Type Culture Collection (ATCC), including Gram-positive bacteria, Gram-negative bacteria, yeasts, and filamentous fungi.

The tested microorganisms were *Enterococcus faecalis* ATCC 19433, *Enterococcus faecium* ATCC 27270, *Staphylococcus epidermidis* ATCC 12228, *Staphylococcus aureus* ATCC 25923, *Escherichia coli* (O157:H7) ATCC 43888, *Salmonella enterica* subsp. *enterica* serovar Typhimurium WDCM 00031 derived from ATCC 14028, *Pseudomonas aeruginosa* ATCC 10145, *Listeria monocytogenes* ATCC 19115, *Aspergillus niger* ATCC 6275, and *Candida albicans* ATCC 10231.

The essential oil was dissolved in dimethyl sulfoxide (DMSO) to obtain a stock solution of 40 mg/mL. Serial two-fold dilutions were prepared in the corresponding culture medium to obtain the tested concentration range, with 2000 µg/mL as the highest concentration evaluated. Mueller–Hinton broth was used for bacterial assays, whereas Sabouraud dextrose broth was used for the yeast and filamentous fungal assays. The microbial suspensions were initially adjusted to a 0.5 McFarland turbidity standard and subsequently diluted in the corresponding culture medium to obtain final inoculum concentrations in the assay wells of 5 × 10^5^ CFU/mL for bacteria, 2.5 × 10^5^ CFU/mL for *Candida albicans*, and 5 × 10^4^ spores/mL for *Aspergillus niger*. The bacterial assays were incubated at 37 °C for 24 h, the yeast assay at 37 °C for 48 h, and the filamentous fungal assay at 28 °C for 48 h.

Ampicillin was used as the positive control for Gram-positive bacteria, ciprofloxacin for Gram-negative bacteria and *Listeria monocytogenes*, and amphotericin B for *A. niger* and *C. albicans*. Wells containing culture medium and the maximum concentration of DMSO were included as negative controls to verify that the solvent did not inhibit microbial growth. The MIC was defined as the lowest concentration of essential oil that visibly inhibited microbial growth under the assay conditions.

#### 3.6.2. Radical Scavenging Assays

The radical-scavenging activity of *H. eriocephala* essential oil was evaluated using the DPPH and ABTS assays, following the procedure described by Cartuche et al. [55], with minor modifications. Each of the three independently distilled essential oil samples was separately dissolved in methanol at 80 mg/mL and subjected to twofold serial dilution. For both assays, 30 µL of each essential oil dilution was mixed with 270 µL of the corresponding radical working solution in a 96-well microplate, resulting in final assay concentrations of 8000, 4000, 2000, 1000, 500, 250, 125, and 62.5 µg/mL in a total reaction volume of 300 µL. Corresponding solvent-control wells contained 30 µL of methanol and 270 µL of the respective radical working solution without essential oil. Trolox was used as the positive reference.

For the DPPH assay, a methanolic solution of 2,2-diphenyl-1-picrylhydrazyl was prepared and adjusted to an absorbance of 1.10 ± 0.02 at 515 nm. The reaction mixtures were incubated for 60 min at room temperature in the dark, and absorbance was measured at 515 nm using an EPOCH 2 microplate reader (BioTek, Winooski, VT, USA).

For the ABTS assay, the ABTS radical cation was generated by mixing equal volumes of 7.4 mM ABTS and 2.6 mM potassium persulfate solutions and maintaining the mixture for 14 h at room temperature in the dark. Before use, the radical solution was diluted with methanol to an absorbance of 1.10 ± 0.02 at 734 nm. The reaction mixtures were incubated for 60 min at room temperature in the dark, and absorbance was measured at 734 nm using the same microplate reader.

Radical-scavenging activity was calculated according to Equation (1):

(1) $$SC\left(\%\right)=\frac{A_{0}-A_{i}}{A_{0}}\times 100$$

where $A_{0}$ is the absorbance of the radical control and $A_{i}$ is the absorbance of the reaction mixture containing the essential oil. The methanol contribution was therefore matched between the control and sample wells. No sample-specific blank containing essential oil without the radical solution was included; consequently, no additional correction for the intrinsic background absorbance of the essential oil was applied.

SC_50_ was defined as the essential oil concentration required to reduce the radical signal by 50%. Concentration–response data were fitted by nonlinear regression using a GraphPad Prism 8.0.1 four-parameter logistic model with variable slope. (GraphPad Software Inc., San Diego, CA, USA). SC_50_ values were calculated independently for each extraction replicate. The three independently distilled essential oil samples were evaluated separately in a single experimental run, and each concentration was measured in technical triplicate. Technical replicates were averaged for each extraction replicate, and results are expressed as mean ± SD across the three independent extraction replicates (n = 3). When 50% scavenging was not reached within the evaluated concentration range, the SC_50_ value was not extrapolated and was reported as greater than the highest concentration tested.

For the ABTS assay, a Trolox calibration curve ranging from 1.25 to 50 µM was generated under the same experimental conditions and fitted by least-squares linear regression. The resulting equation was y = −0.02066X + 1.152, with R^2^ = 0.9955. The Trolox-equivalent concentration corresponding to the essential oil response was obtained by interpolation from the calibration curve and normalized to the mass of essential oil evaluated. TEAC was calculated according to Equation (2):

(2) $$TEAC\;\left(\mu M\;TE/g\;EO\right)=\frac{C_{TE}}{m_{EO}}$$

where $C_{TE}$ is the Trolox-equivalent obtained from the calibration curve (µM), and $m_{EO}$ is the mass of essential oil evaluated in the reaction mixture (g).

#### 3.6.3. Cholinesterase Inhibitory Assay

The inhibitory activity of *H. eriocephala* essential oil against acetylcholinesterase (AChE) and butyrylcholinesterase (BuChE) was evaluated in vitro using a modified Ellman microplate assay [56]. AChE from *Electrophorus electricus* (Sigma-Aldrich, C3389, St. Louis, MO, USA) and BuChE from equine serum (Sigma-Aldrich, SRE020, St. Louis, MO, USA) were used. The working activity of both enzyme solutions was 0.5 U/mL.

For each assay, 40 µL of phosphate-buffered saline (PBS, pH 7.4), 100 µL of 3 mM DTNB solution, 20 µL of the corresponding 15 mM substrate solution, and 20 µL of the essential oil dilution were added to each well. Acetylthiocholine iodide and butyrylthiocholine iodide were used as substrates for AChE and BuChE, respectively. The mixture was preincubated for 3 min at 25 °C, after which 20 µL of the corresponding enzyme solution was added to initiate the reaction. The final reaction volume was 200 µL per well, corresponding to final concentrations of 1.5 mM for DTNB and 1.5 mM for the respective substrate. Absorbance was monitored at 412 nm for 60 min using an EPOCH 2 microplate reader (BioTek, Winooski, VT, USA).

Each independently distilled essential oil sample was dissolved in HPLC-grade methanol at 10 mg/mL and subjected to a tenfold serial dilution to obtain four working concentrations. The working solutions were prepared at 10, 1, 0.1, and 0.01 mg/mL, corresponding to final assay concentrations of 1000, 100, 10, and 1 µg/mL, respectively, after addition of 20 µL to the 200 µL reaction mixture. Methanol at the same final proportion used in the sample wells was included as the solvent control, and donepezil hydrochloride was used as the positive reference inhibitor.

A single experimental run was performed for each enzyme. The three independently distilled essential oil samples were evaluated separately, and each concentration was measured in technical triplicate. Technical replicates were averaged for each extraction replicate. IC_50_ values were calculated by nonlinear regression using GraphPad Prism 8.0.1 (GraphPad Software, San Diego, CA, USA) and are expressed as mean ± SD across the three independent extraction replicates (n = 3).

## 4. Conclusions

This study provides the first phytochemical and biological baseline for *Hyptis eriocephala* essential oil collected at Villonaco, southern Ecuador. Sixty-five volatile constituents were tentatively identified, and the relative GC–FID profile was dominated by hydrocarbon sesquiterpenes, particularly α-copaene. The oil showed narrow in vitro antibacterial activity, mainly against *Enterococcus faecium*, an assay-dependent radical-scavenging response limited to ABTS, and preferential but moderate BuChE inhibition. These findings should be considered preliminary because they derive from material collected at a single location and period and from in vitro screening assays. They do not establish a species-wide chemotype, mechanism of action, or therapeutic potential. Broader geographic, seasonal, and activity-guided studies are required to assess the reproducibility and biological relevance of this profile.

## Figures and Tables

**Table 1:** Yield and physicochemical properties of *Hyptis eriocephala* essential oil.

Property	Mean ± SD
Yield (%)	0.13 ± 0.04
Refractive index, *n*^20^_D_	1.4875 ± 0.0020
Specific optical rotation [*α*]^20^_D_ (°)	−11.05 ± 0.03

Note: Yield was calculated on a dry-weight basis. Refractive index and specific optical rotation were measured at 20 °C; optical rotation was determined after dilution in dichloromethane. Values are expressed as mean ± SD across three independently distilled essential oil samples (n = 3).

**Table 2:** Volatile constituents of *Hyptis eriocephala* essential oil tentatively identified by GC–MS and their relative peak-area composition determined by GC–FID.

No.	Retention Time (min)	Compound	LRI_cal_	LRI_ref_	Relative GC–FID Peak Area (%)	Molecular Formula
1	8.640	α-thujene	926	924	0.06 ± 0.01	C_10_H_16_
2	8.932	α-pinene	933	932	6.98 ± 0.03	C_10_H_16_
3	9.623	camphene	950	946	0.01 ± 0.00	C_10_H_16_
4	10.623	sabinene	973	969	0.72 ± 0.00	C_10_H_16_
5	10.813	β-pinene	978	974	2.35 ± 0.01	C_10_H_16_
6	11.401	myrcene	992	988	1.37 ± 0.01	C_10_H_16_
7	12.014	p-mentha-1(7),8-diene	1006	1003	0.21 ± 0.00	C_10_H_16_
8	12.122	δ-3-carene	1008	1008	0.45 ± 0.00	C_10_H_16_
9	12.622	α-terpinene	1018	1014	0.03 ± 0.00	C_10_H_16_
10	12.993	o-cymene	1026	1022	0.23 ± 0.00	C_10_H_14_
11	13.204	limonene	1030	1024	5.44 ± 0.05	C_10_H_16_
12	13.255	β-phellandrene	1031	1025	4.96 ± 0.02	C_10_H_16_
13	13.581	(Z)-β-ocimene	1038	1032	0.14 ± 0.00	C_10_H_16_
14	14.071	(E)-β-ocimene	1049	1044	0.06 ± 0.00	C_10_H_16_
15	14.588	γ-terpinene	1059	1054	0.03 ± 0.01	C_10_H_16_
16	15.881	p-mentha-2,4(8)-diene	1087	1085	1.30 ± 0.01	C_10_H_16_
17	16.177	p-cymenene	1093	1089	0.03 ± 0.01	C_10_H_12_
18	16.710	linalool	1104	1095	0.07 ± 0.00	C_10_H_18_O
19	16.918	nonanal	1108	1100	0.06 ± 0.00	C_9_H_18_O
20	20.503	terpinen-4-ol	1183	1174	0.15 ± 0.00	C_10_H_18_O
21	20.890	p-cymen-8-ol	1191	1179	0.08 ± 0.00	C_10_H_14_O
22	21.792	decanal	1210	1201	0.05 ± 0.01	C_10_H_20_O
23	25.366	isobornyl acetate	1286	1283	0.14 ± 0.00	C_12_H_20_O_2_
24	28.128	α-cubebene	1348	1348	6.70 ± 0.06	C_15_H_24_
25	29.090	isoledene	1369	1374	0.24 ± 0.00	C_15_H_24_
26	29.393	α-copaene	1376	1374	28.20 ± 0.24	C_15_H_24_
27	29.716	β-bourbonene	1383	1387	0.98 ± 0.01	C_15_H_24_
28	29.927	β-cubebene	1388	1387	1.28 ± 0.01	C_15_H_24_
29	30.005	β-elemene	1390	1389	0.02 ± 0.00	C_15_H_24_
30	30.737	α-gurjunene	1407	1409	0.58 ± 0.01	C_15_H_24_
31	31.257	(E)-caryophyllene	1419	1417	5.31 ± 0.05	C_15_H_24_
32	31.699	β-copaene	1429	1430	0.56 ± 0.01	C_15_H_24_
33	31.954	α-guaiene	1435	1437	0.18 ± 0.00	C_15_H_24_
34	32.321	aromadendrene	1444	1439	0.16 ± 0.00	C_15_H_24_
35	32.577	geranyl acetone	1450	1453	0.66 ± 0.01	C_13_H_22_O
36	32.777	α-humulene	1455	1452	1.90 ± 0.02	C_15_H_24_
37	32.961	allo-aromadendrene	1459	1458	0.25 ± 0.00	C_15_H_24_
38	33.624	γ-muurolene	1475	1478	1.00 ± 0.01	C_15_H_24_
39	33.862	germacrene D	1481	1480	9.29 ± 0.09	C_15_H_24_
40	34.175	β-selinene	1488	1489	0.13 ± 0.01	C_15_H_24_
41	34.318	δ-selinene	1491	1492	0.20 ± 0.00	C_15_H_24_
42	34.355	trans-muurola-4(14),5-diene	1492	1493	0.34 ± 0.00	C_15_H_24_
43	34.495	γ-amorphene	1496	1495	1.47 ± 0.02	C_15_H_24_
44	34.610	valencene	1498	1496	0.06 ± 0.00	C_15_H_24_
45	34.746	bicyclogermacrene	1502	1500	0.02 ± 0.01	C_15_H_24_
46	34.937	(E,E)-α-farnesene	1506	1505	0.37 ± 0.00	C_15_H_24_
47	35.192	δ-amorphene	1513	1511	0.07 ± 0.01	C_15_H_24_
48	35.423	δ-cadinene	1519	1522	6.03 ± 0.07	C_15_H_24_
49	35.552	cis-calamenene	1522	1528	0.49 ± 0.00	C_15_H_22_
50	35.978	trans-cadina-1,4-diene	1533	1533	0.47 ± 0.00	C_15_H_24_
51	36.141	α-cadinene	1537	1537	0.30 ± 0.00	C_15_H_24_
52	36.328	α-calacorene	1541	1544	0.31 ± 0.01	C_15_H_20_
53	37.012	germacrene B	1558	1559	0.95 ± 0.01	C_15_H_24_
54	37.202	(E)-nerolidol	1563	1561	0.10 ± 0.00	C_15_H_26_O
55	37.760	spathulenol	1577	1577	0.21 ± 0.03	C_15_H_24_O
56	37.950	caryophyllene oxide	1582	1582	0.53 ± 0.01	C_15_H_24_O
57	38.447	viridiflorol	1594	1592	0.10 ± 0.03	C_15_H_26_O
58	38.848	ledol	1605	1602	0.36 ± 0.03	C_15_H_26_O
59	39.066	widdrol	1610	1599	0.23 ± 0.07	C_15_H_26_O
60	39.450	dillapiole	1621	1620	0.08 ± 0.05	C_12_H_14_O_4_
61	39.756	1-epi-cubenol	1629	1627	0.31 ± 0.00	C_15_H_26_O
62	39.998	cis-cadin-4-en-7-ol	1635	1635	0.16 ± 0.00	C_15_H_26_O
63	40.314	α-muurolol	1643	1644	0.30 ± 0.00	C_15_H_26_O
64	40.831	α-cadinol	1657	1652	0.19 ± 0.01	C_15_H_26_O
65	49.905	(5E,9Z)-farnesyl acetone	1885	1886	0.78 ± 0.01	C_18_H_30_O
		Hydrocarbon monoterpenes			24.36	
		Oxygenated monoterpenes			0.35	
		Hydrocarbon sesquiterpenes			67.88	
		Oxygenated sesquiterpenes			2.49	
		Others			1.73	
		Total identified			96.81	

Notes: LRIcal, calculated linear retention index on the TR-5MS column; LRIref, reference value reported in the literature or database. Relative percentages were calculated by GC–FID peak-area normalization without an internal standard, calibration curves, or response correction factors. Values are expressed as mean ± SD across three independent extraction replicates (n = 3) and represent relative peak-area composition rather than absolute concentrations.

**Table 3:** Minimum inhibitory concentrations of *Hyptis eriocephala* essential oil and reference antimicrobials against the tested bacterial, yeast and filamentous fungal strains.

Microorganism	*H. eriocephala* Essential Oil MIC (µg/mL)	Reference Antimicrobial MIC (µg/mL)
Gram-positive cocci		Ampicillin (1 mg/mL)
*Enterococcus faecalis* ATCC^®^ 19433	2000	0.7812
*Enterococcus faecium* ATCC^®^ 27270	250	<0.3906
*Staphylococcus epidermidis* ATCC^®^ 12228	>2000	<0.3906
*Staphylococcus aureus* ATCC^®^ 25923	>2000	<0.3906
Rod-shaped bacteria		Ciprofloxacin (1 mg/mL)
*Escherichia coli* (O157:H7) ATCC^®^ 43888	>2000	1.5625
*Salmonella enterica* subsp. *enterica* serovar Typhimurium WDCM 00031, derived ATCC^®^ 14028	>2000	<0.3906
*Pseudomonas aeruginosa* ATCC^®^ 10145	>2000	<0.3906
*Listeria monocytogenes* ATCC^®^ 19115	>2000	1.5625
Yeasts and filamentous fungus		Amphotericin B (250 µg/mL)
*Aspergillus niger* ATCC^®^ 6275	>2000	<0.098
*Candida albicans* ATCC^®^ 10231	>2000	<0.098

Note: MIC values are expressed in µg/mL. Ampicillin, ciprofloxacin, and amphotericin B were used as positive controls; DMSO (5%, *v*/*v*) was used as the solvent control. Values >2000 indicate no inhibition at the highest concentration tested.

**Table 4:** DPPH and ABTS radical-scavenging activity of *Hyptis eriocephala* essential oil.

Essential Oil	DPPH SC_50_	ABTS SC_50_	TEAC
	(µg/mL)		(µM TE/g EO)
*H. eriocephala*	>8000	76.87 ± 1.05	30.06 ± 1.16
Trolox ^a^	35.54 ± 1.04 µM	29.09 ± 1.05	—

Note: SC_50_ values are expressed in µg/mL for the essential oil and in µM for Trolox. TEAC is expressed as µM Trolox equivalents per gram of essential oil (µM TE/g EO). Values for the essential oil are expressed as mean ± SD across three independent extraction replicates (n = 3), each measured in technical triplicate. The value >8000 indicates that 50% DPPH scavenging was not reached and that no SC_50_ value was extrapolated; —, not applicable. ^a^ Trolox was used as the reference antioxidant.

## Data Availability

The data presented in this study are available within the article.

## References

[1] Atanasov A.G., Waltenberger B., Pferschy-Wenzig E.-M., Linder T., Wawrosch C., Uhrin P., Temml V., Wang L., Schwaiger S., Heiss E.H. (2015). Discovery and Resupply of Pharmacologically Active Plant-Derived Natural Products: A Review. Biotechnol. Adv..

[2] Chaachouay N., Zidane L. (2024). Plant-Derived Natural Products: A Source for Drug Discovery and Development. Drugs Drug Candidates.

[3] Jansen C., Baker J.D., Kodaira E., Ang L., Bacani A.J., Aldan J.T., Shimoda L.M.N., Salameh M., Small-Howard A.L., Stokes A.J. (2021). Medicine in Motion: Opportunities, Challenges and Data Analytics-Based Solutions for Traditional Medicine Integration into Western Medical Practice. J. Ethnopharmacol..

[4] Wainwright C.L., Teixeira M.M., Adelson D.L., Buenz E.J., David B., Glaser K.B., Harata-Lee Y., Howes M.J.R., Izzo A.A., Maffia P. (2022). Future Directions for the Discovery of Natural Product-Derived Immunomodulating Drugs: An IUPHAR Positional Review. Pharmacol. Res..

[5] Mungwari C.P., King’ondu C.K., Sigauke P., Obadele B.A. (2025). Conventional and Modern Techniques for Bioactive Compounds Recovery from Plants: Review. Sci. Afr..

[6] Bunse M., Daniels R., Gründemann C., Heilmann J., Kammerer D.R., Keusgen M., Lindequist U., Melzig M.F., Morlock G.E., Schulz H. (2022). Essential Oils as Multicomponent Mixtures and Their Potential for Human Health and Well-Being. Front. Pharmacol..

[7] Bakkali F., Averbeck S., Averbeck D., Idaomar M. (2008). Biological Effects of Essential Oils—A Review. Food Chem. Toxicol..

[8] de la Torre L., Navarrete H., Muriel M.P., Macía M.J., Balslev H., Herbario QCA de la Escuela de Ciencias Biológicas de la Pontificia Universidad Católica del Ecuador, Herbario AAU del Departamento de Ciencias Biológicas de la Universidad de Aarhus (2008). Enciclopedia de Las Plantas Útiles Del Ecuador.

[9] Morocho V., Benitez Á., Carrión B., Cartuche L. (2024). Novel Study on Chemical Characterization and Antimicrobial, Antioxidant, and Anticholinesterase Activity of Essential Oil from Ecuadorian Bryophyte *Syzygiella rubricaulis* (Nees) Stephani. Plants.

[10] Frey M., Gohr S.T., Köllner T.G., Bathe U., Lackus N.D., Padilla-Gonzalez F., Ro D.K., O’Connor S.E., Degenhardt J., Tissier A. (2025). Biosynthesis of Biologically Active Terpenoids in the Mint Family (Lamiaceae). Nat. Prod. Rep..

[11] Sun M., Zhang Y., Zhu L., Liu N., Bai H., Sun G., Zhang J., Shi L. (2022). Chromosome-Level Assembly and Analysis of the Thymus Genome Provide Insights into Glandular Secretory Trichome Formation and Monoterpenoid Biosynthesis in Thyme. Plant Commun..

[12] Moshari-Nasirkandi A., Alirezalu A., Alipour H., Amato J. (2023). Screening of 20 Species from Lamiaceae Family Based on Phytochemical Analysis, Antioxidant Activity and HPLC Profiling. Sci. Rep..

[13] Ramos Da Silva L.R., Ferreira O.O., Cruz J.N., De Jesus Pereira Franco C., Oliveira Dos Anjos T., Cascaes M.M., Almeida Da Costa W., Helena De Aguiar Andrade E., Santana De Oliveira M. (2021). *Lamiaceae* Essential Oils, Phytochemical Profile, Antioxidant, and Biological Activities. Evid. Based Complement. Altern. Med..

[14] Harley R.M. (1988). Revision of Generic Limits in *Hyptis* Jacq. (Labiatae) and Its Allies. Bot. J. Linn. Soc..

[15] Franco C.R.P., Alves P.B., Andrade D.M., de Jesus H.C.R., Silva E.J.S., Santos E.A.B., Antoniolli Â.R., Quintans-Júnior L.J. (2011). Essential Oil Composition and Variability in Hyptis Fruticosa. Rev. Bras. Farmacogn..

[16] Dossa A.F., Fassinou Hotegni N.V., N’Danikou S., Yayi-Ladekan E., Adjé C.A.O., Lagnika L., Bokonon-Ganta A.H., Achigan-Dako E.G. (2024). Genetic Diversity, Essential Oil’s Chemical Constituents of Aromatic Plant *Mesosphaerum suaveolens* (L.) Kuntze Syn. *Hyptis suaveolens* (L.) Poit. and Its Uses in Crop Protection: A Review. Front. Plant Sci..

[17] Grassi P., Nuñez M.J., Reyes T.S.U., Franz C. (2008). Chemical Variation in the Essential Oil Composition of *Hyptis suaveolens* (L.) Poit. (Lamiaceae). Nat. Prod. Commun..

[18] Figueiredo A.C., Barroso J.G., Pedro L.G., Scheffer J.J.C. (2008). Factors Affecting Secondary Metabolite Production in Plants: Volatile Components and Essential Oils. Flavour Fragr. J..

[19] Sonigra P., Meena M. (2025). Influence of *Hyptis suaveolens* Growth Stage on Essential Oil Composition, Antimicrobial and Antioxidant Activities. Sci. Rep..

[20] McNeil M., Facey P., Porter R. (2011). Essential Oils from the Hyptis Genus—A Review (1909-2009). Nat. Prod. Commun..

[21] Lima M.N.N.D., Costa J.S.D., Guimarães B.A., Freitas J.J.S., Setzer W.N., Silva J.K.R.D., Maia J.G.S., Figueiredo P.L.B., Nancy M., De Lima N. (2023). Chemometrics of the Composition and Antioxidant Capacity of *Hyptis crenata* Essential Oils from Brazil. Molecules.

[22] Lima A.S., Fernandes Y.M.L., Silva C.R., Costa-Junior L.M., Figueiredo P.L.B., Monteiro O.S., Maia J.G.S., da Rocha C.Q. (2022). Anthelmintic Evaluation and Essential Oils Composition of *Hyptis dilatata* Benth. and *Mesosphaerum suaveolens* Kuntze from the Brazilian Amazon. Acta Trop..

[23] Werker E. (1993). Function of Essential Oil-Secreting Glandular Hairs in Aromatic Plans of Lamiacea—A Review. Flavour Fragr. J..

[24] Nejad Ebrahimi S., Hadian J., Mirjalili M.H., Sonboli A., Yousefzadi M. (2008). Essential Oil Composition and Antibacterial Activity of *Thymus caramanicus* at Different Phenological Stages. Food Chem..

[25] Noudogbessi J., Agbangnan P., Yehouenou B., Adjalian E., Nonviho G., Osseni M.A., Wotto V., Figuérédo G., Chalchat J., Sohounhloue D. (2013). Physico-Chemical Properties of *Hyptis suaveolens* Essential Oil. Int. J. Med. Aromat. Plants.

[26] Valarezo E., Toledo-Ruiz L., Coque-Saetama W., Caraguay-Martínez A., Jaramillo-Fierro X., Cumbicus N., Meneses M.A. (2025). Chemical Composition, Enantiomeric Distribution and Antimicrobial, Antioxidant and Antienzymatic Activities of Essential Oil from Leaves of *Citrus* x *limonia*. Molecules.

[27] Babushok V.I., Linstrom P.J., Zenkevich I.G. (2011). Retention Indices for Frequently Reported Compounds of Plant Essential Oils. J. Phys. Chem. Ref. Data.

[28] Bizzo H.R., Brilhante N.S., Nolvachai Y., Marriott P.J. (2023). Use and Abuse of Retention Indices in Gas Chromatography. J. Chromatogr. A.

[29] Cagliero C., Bicchi C., Marengo A., Rubiolo P., Sgorbini B. (2022). Gas Chromatography of Essential Oil: State-of-the-Art, Recent Advances, and Perspectives. J. Sep. Sci..

[30] Souleymane B., Kanigui S., Raphaël O.K., Alexis G.L., Jacques K.D., Mohamed B.B. (2024). Essential Oil of *Hyptis suaveolens* (L.) Poit: A Comprehensive Study of Its Chemical and Biological Properties for Therapeutic and Industrial Applications. GSC Biol. Pharm. Sci..

[31] Feitosa-Alcantara R.B., Bacci L., Blank A.F., Alves P.B., Silva I.M.D.A., Soares C.A., Sampaio T.S., Nogueira P.C.D.L., Arrigoni-Blank M.D.F. (2017). Essential Oils of *Hyptis pectinata* Chemotypes: Isolation, Binary Mixtures and Acute Toxicity on Leaf-Cutting Ants. Molecules.

[32] Nantitanon W., Chowwanapoonpohn S., Okonogi S. (2007). Antioxidant and Antimicrobial Activities of *Hyptis suaveolens* Essential Oil. Sci. Pharm..

[33] Kerdudo A., Njoh Ellong E., Gonnot V., Boyer L., Michel T., Adenet S., Rochefort K., Fernandez X. (2016). Essential Oil Composition and Antimicrobial Activity of *Hyptis atrorubens* Poit. from Martinique (F.W.I.). J. Essent. Oil Res..

[34] Santos P.O., Costa M.D.J.C., Alves J.A.B., Nascimento P.F.C., De Melo D.L.M., Barbosa A.M., Trindade R.D.C., Blank A.F., Arrigoni-Blank M.F., Alves P.B. (2008). Chemical Composition and Antimicrobial Activity of the Essential Oil of Hyptis Pectinata (l.) Poit. Quim. Nova.

[35] Susanti Y., A’yun A.Q. (2024). Phytochemical, Antioxidant, and Antibacterial Activity of Essential Oil Hyptis Capitata Using Solvent-Free Microwave Extraction. J. Appl. Agric. Sci. Technol..

[36] Morocho V., Eras O., Rojas T., Jiménez B., Roa M.F., Cartuche L. (2025). Biological Activity and Chemical Composition of Essential Oil from Leaves and Fruits of *Zanthoxylum mantaro* (J.F.Macbr.) J.F.Macbr. Antibiotics.

[37] Calva J., Cartuche L., Castillo L.N., Morocho V. (2023). Biological Activities and Chemical Composition of Essential Oil from *Hedyosmum purpurascens* (Todzia)—An Endemic Plant in Ecuador. Molecules.

[38] Flores M., Rojas L., Aparicio R., Lucena M.E., Usubillaga A. (2015). Essential Oil Composition and Antibacterial Activity of *Hyptis colombiana* from the Venezuelan Andes. Nat. Prod. Commun..

[39] Caesar L.K., Cech N.B. (2019). Synergy and Antagonism in Natural Product Extracts: When 1 + 1 Does Not Equal 2. Nat. Prod. Rep..

[40] Shahidi F., Samarasinghe A. (2025). How to Assess Antioxidant Activity? Advances, Limitations, and Applications of in Vitro, in Vivo, and Ex Vivo Approaches. Food Prod. Process. Nutr..

[41] Tafurt-García G., Jiménez-Vidal L.F., Calvo-Salamanca A.M. (2015). Antioxidant capacity and total phenol content of *Hyptis* spp., *P. heptaphyllum*, *T. panamensis*, *T. rhoifolia*, and *Ocotea* sp.. Rev. Colomb. Quím..

[42] Beltrán S.B., Sierra L.J., Fernández-Alonso J.L., Romero A.K., Martínez J.R., Stashenko E.E. (2023). Antioxidant Properties and Secondary Metabolites Profile of Hyptis Colombiana at Various Phenological Stages. Molecules.

[43] Bonesi M., Menichini F., Tundis R., Loizzo M.R., Conforti F., Passalacqua N.G., Statti G.A., Menichini F. (2010). Acetylcholinesterase and Butyrylcholinesterase Inhibitory Activity of Pinus Species Essential Oils and Their Constituents. J. Enzym. Inhib. Med. Chem..

[44] Jaramillo D., Calva J., Bec N., Larroque C., Vidari G., Armijos C. (2022). Chemical Characterization and Biological Activity of the Essential Oil from Araucaria Brasiliensis Collected in Ecuador. Molecules.

[45] Panamito M.F., Bec N., Valdivieso V., Salinas M., Calva J., Ramírez J., Larroque C., Armijos C. (2021). Chemical Composition and Anticholinesterase Activity of the Essential Oil of Leaves and Flowers from the Ecuadorian Plant *Lepechinia paniculata* (Kunth) Epling. Molecules.

[46] Chen W.N., Chin K.W., Tang K.S., Agatonovic-Kustrin S., Yeong K.Y. (2023). Neuroprotective, Neurite Enhancing, and Cholinesterase Inhibitory Effects of Lamiaceae Family Essential Oils in Alzheimer’s Disease Model. J. Herb. Med..

[47] Orhan I., Şener B., Kartal M., Kan Y. (2008). Activity of Essential Oils and Individual Components against Acetyl- and Butyrylcholinesterase. Z. Naturforsch. C J. Biosci..

[48] Savelev S.U., Okello E.J., Perry E.K. (2004). Butyryl- and Acetyl-Cholinesterase Inhibitory Activities in Essential Oils of Salvia Species and Their Constituents. Phytother. Res..

[49] Flores M.E., Arias G.I. (2024). Influencia de La Variabilidad Climática En Temperatura, Precipitación y Velocidad Del Viento En Villonaco, Ecuador. Rev. Cienc. UNEMI.

[50] (1998). Standard: Refractive Index at 20 °C—Reference Method.

[51] (1998). Huiles Essentielles—Détermination Du Pouvoir Rotatoire.

[52] Adams R.P. (2007). Identification of Essential Oil Components by Gas Chromatography/Mass Spectrometry.

[53] Linstrom P.J., Mallard W.G. NIST Chemistry WebBook.

[54] van Den Dool H., Kratz P.D. (1963). A Generalization of the Retention Index System Including Linear Temperature Programmed Gas—Liquid Partition Chromatography. J. Chromatogr. A.

[55] Cartuche L., Vallejo C., Castillo E., Cumbicus N., Morocho V. (2024). Chemical Profiling of *Drimys granadensis* (Winteraceae) Essential Oil, and Their Antimicrobial, Antioxidant, and Anticholinesterase Properties. Plants.

[56] Ellman G.L., Courtney K.D., Andres V., Featherstone R.M. (1961). A New and Rapid Colorimetric Determination of Acetylcholinesterase Activity. Biochem. Pharmacol..

